# Retrospective Observational Study on Implant Site Preparation Using Magnetodynamic Surgery vs. Piezoelectric and Traditional Surgery

**DOI:** 10.3390/jcm14248841

**Published:** 2025-12-14

**Authors:** Lorenzo Bevilacqua, Luca De Angelis, Lucio Torelli, Antonio Scarano, Gianmarco Gronelli, Michele Maglione

**Affiliations:** 1 University Clinical Department of Medical, Surgical and Health Sciences, University of Trieste, 34129 Trieste, TS, Italy; l.bevilacqua@fmc.units.it (L.B.); torelli@units.it (L.T.);; 2 Department of Innovative Technologies in Medicine and Dentistry, University of Chieti, 66100 Chieti, CH, Italy; ascarano@unich.it

**Keywords:** piezosurgery, magnetic mallet, drills, implant, oral surgery

## Abstract

**Objective:** This study compared magnetodynamic surgery, traditional drill-based surgery, and piezoelectric surgery for the preparation of the implant site, focusing on operative time and intra/postoperative discomfort. **Methods:** A total of 86 patients (69.8% female, 30.2% male) treated at the Oral Surgery Clinic, University of Trieste, were included: 43 underwent implant placement with the Magnetic Mallet (MM); the remaining 43 received preparations with the Piezodevice (IP) on one side and drills (Ds) on the other. All surgeries were performed by the same operator. Data included bone quality, operative time, and postoperative questionnaire responses for pain (VAS) and analgesic use. A statistical analysis was conducted using Mann–Whitney U and Kruskal–Wallis tests. **Results:** Significant differences emerged in operative times and pain perception, influenced by bone quality. The MM and D had comparable times in D1–D2 and D3–D4 bone, but the D produced higher VAS scores. The MM vs. IP showed significant differences in absolute times (*p* = 0.00018) and relative times for both D1–D2 (*p* = 0.01875) and D3–D4 (*p* = 0.00584), with qualitative VAS differences. The IP vs. D also showed significant absolute (*p* = 0.000005) and relative time differences for D1–D2 (*p* = 0.00718) and D3–D4 (*p* = 0.000145), with VAS variations. In the MM group, higher bone density significantly prolonged times (*p* = 0.04136). **Conclusions:** Within the limits of this study, the traditional drill-based technique remains valid and widely used, but the Magnetic Mallet can offer advantages in terms of patient comfort and postoperative recovery. The Piezodevice, while excelling in tissue preservation, is limited by longer operative times.

## 1. Introduction

The need to explore and implement surgical techniques that are as minimally invasive and traumatic as possible [1] has driven researchers and clinicians to introduce new tools in the field of Oral Surgery. These innovations aim to reduce inflammation, minimize secondary bone resorption caused by surgical procedures, and promote tissue healing [2]. The use of a dedicated set of drills mounted on a slow-speed handpiece for implant site preparation is undoubtedly a well-established approach today, especially among experienced practitioners. However, this method is not without its drawbacks. These are primarily related to the overheating of the bone tissue [3,4,5], potential damage to soft tissues and vital structures [6], as well as the generation of macro-vibrations [7], which can compromise the precision of the procedure. Both piezoelectric and magnetodynamic instruments represent modern alternatives to traditional drills for implant site preparation. They share fundamental advantages centered on minimizing tissue trauma and maximizing operative control. Both technologies facilitate a more conservative, less traumatic approach to bone tissue. Piezoelectric instruments achieve this through micrometric and selective cutting [8], which protects soft tissues [9,10,11]. Magnetodynamic technology achieves a similar effect with the application of controlled, high-intensity, and standardized forces to the bone tissue [12], minimizing the impact time between the insert and the tissue to just 80 µs, enabling precise site preparation and actively preventing over-preparation [13]. Both methods contribute to better outcomes at the surgical site. The Magnetic Mallet uniquely enables bone compaction [12], which can improve the stability of the implant site. Both minimize the risk of bone overheating, a major concern with traditional drills. The piezoelectric instrument requires tip irrigation for cooling, cleaning, and debris removal via the cavitation effect [14,15]. Conversely, the minimal contact time of the magnetodynamic insert eliminates heat generation, thus requiring no irrigation [13]. The absence of irrigation in magnetodynamic surgery significantly enhances the operator’s visibility [13] and reduces inflammation [2], with a lower risk of developing benign paroxysmal positional vertigo [16]. Furthermore, the reduced trauma and inflammation associated with a conservative approach [2] and the greater precision [17] of both instruments can establish these technologies as valid alternatives to conventional methods. The main disadvantages of the piezoelectric instrument are the longer operating time and faster wear of the inserts [18].

The existing literature often focuses on comparing only two techniques (e.g., traditional vs. piezoelectric) [19,20] or concentrates on biomechanical outcomes [2]. There is a distinct lack of studies that integrate operational efficiency with the patient’s perspective across all three methods simultaneously.

The aim of this study is therefore to address this literature gap by examining whether there are differences in implant site preparation by comparing the traditional method, the piezoelectric instrument, and the Magnetic Mallet. This study compared implant site preparation times, both in absolute terms and relative to the bone quality at the surgical site, for each surgical method. It also focused on the subjective evaluation of intraoperative and postoperative pain, as well as the need for postoperative analgesic therapy.

The recommendation for strengthening the reporting of observational studies in epidemiology, STROBE, and the template for intervention descriptions and replications (TiDieR) were followed.

This study observed the good clinical practice (CPMP/ICH/135/95) guidelines and was approved by the ethics committee of the University of Trieste (n. 88-10.05.2018).

Informed consent was obtained from every patient for the clinical procedure and their enrollment in this study.

## 2. Materials and Methods

### 2.1. Study Design

This retrospective study analyzed the clinical outcome of 86 patients, all treated at the Oral Surgery Unit (Department of Medical Sciences, Surgery and Health, University of Trieste, Italy). Of these, 43 patients—30 women (69.8%) and 13 men (30.2%), aged between 45 and 70 years—underwent implant placement using magnetodynamic surgery, with a total of 84 implants placed by the same operator. The collected data were compared with a “control” group consisting of 43 patients, 30 women (69.8%) and 13 men (30.2%), where each patient underwent the placement of at least two implants in contralateral sites: at least one implant site was prepared using the “Piezodevice supplied by Esacrom^®^ (Imola, Italy),” while the contralateral site was prepared using a micromotor and a specific set of drills, resulting in a total of 150 implants placed, 75 for each method, all by the same operator.

Patients were randomly assigned to the various methodologies. A blind randomized procedure (closed envelope system) was used to assign the patients to the site to be used for piezoelectric technology or traditional preparation with burs. The remaining patients were assigned to the Magnetic Mallet group.

The following study included adult patients undergoing implant therapy with the insertion of conical implants with a diameter between 3.8 and 4.5 mm with a maximum torque of 35 Ncm in the period between January 2013 and October 2024.

The exclusion criteria for this study are indicated in Table 1.

In the preoperative phase, the operator performed CBCT (Cone Beam Computed Tomography) to assess the bone quality and quantity at the surgical site; intraoperatively, the operator recorded the bone quality by evaluating the resistance offered by the bone tissue during drilling, distinguishing the bone into a D1–D2 group, rich in cortical bone, and a D3–D4 group, with a predominant medullary component, according to the Misch classification [21]. The method that was considered to be the reference was the CBCT analysis. The analyzed sample consists of 23 patients in the D1–D2 group and 20 patients in the D3–D4 group. Before the surgery, patients received antibiotic therapy with amoxicillin at a dosage of 2 g one hour prior to the procedure; additionally, patients were asked to perform a mouth rinse with a chlorhexidine solution at 0.2% for 60 s before the intervention. For anesthetizing the area to be treated, an anesthetic containing 2% mepivacaine hydrochloride with a vasoconstrictor at a concentration of 1:100,000 was used. The preparation of the implant site with the conventional technique was carried out using drills mounted on a micromotor, following the protocol provided by the manufacturer of the implant system WINSIX^®^ (BioSAF IN S.r.l., Trezzano Rosa, Milan, Italy). The drills used have the following diameters: TT1L D: 2 mm, TT2L D: 2.2/2.6 mm, TT3L D: 2.6/3 mm, TT4L D: 3.0/3.4 mm, and TT5L D: 3.4/3.8 mm.

For the preparation of the implant site using piezosurgery, the instrument used was the “Surgysonic II (Esacrom^®^ S.r.l., Imola, Italy)” with a sequence of ultrasonic inserts consisting of 5–6 inserts to achieve the desired implant site diameter: the first two inserts, with a pointed shape (ES012X and ES052XG), were used to facilitate the preparation of the implant tunnel, followed by the use of 3–4 inserts (ES040 D: 2.75 mm, ES041 D: 3 mm, ES043 D: 3.35 mm, ES044 D: 3.55 mm) of increasing diameter for the preparation and finalization of the site. The inserts were used according to the manufacturer’s instructions, with abundant irrigation.

For the preparation of the implant site using magnetodynamic surgery, the Magnetic Mallet device (Meta Ergonomica S.r.l., Milan, Italy) was used, with a sequence of specific inserts of increasing diameter. The forces used in our study were type 2 (90 daN) and type 3 (130 daN).

The osteotomes used in sequence were as follows:

- 100-P: Pointed osteotome serving as a pilot insert; the tip has a diameter of 1 mm;
- 160-F: Osteotome with a flat tip and an apical diameter of 1.6 mm;
- 230-F: Osteotome with an apical diameter of 2.3 mm;
- 300-F: Osteotome with an apical diameter of 3 mm;
- 360-F: Osteotome with an apical diameter of 3.6 mm.

The last four inserts were used in sequence to progressively widen the implant site, necessary for the placement of the implant fixture.

The time required for implant site preparation was measured, starting from the preparation of the flap up to the insertion of the implant. Conical implants with a diameter between 3.8 and 4.5 mm with a maximum torque of 35 Ncm were inserted in the prepared site, a cover screw was placed, and the flaps were closed with primary intention using resorbable sutures made of polyglactin 910. In the postoperative period, each patient was prescribed mouth rinses containing 0.2% chlorhexidine to be used twice a day for 14 days and 1 g of paracetamol to be taken in case of pain, with a maximum of 3 tablets per day.

### 2.2. Questionnaire

The questionnaire is a standardized patient clinical diary, identical for all patients, designed to be completed after the surgery.

The questionnaire was utilized to enable the subjective evaluation of pain symptoms and to record the patient’s use of analgesic therapy (1 g of paracetamol) in the days following the procedure.

On the day of the intervention (Day 0), the patient was asked to indicate the level of discomfort caused by the surgical procedure on a Visual Analog Scale ranging from “no discomfort” (0) to “severe discomfort” (10), to report the level of pain perceived in the surgical area, and to specify the number of pain relief tablets taken that day. For pain assessment, the VAS (Visual Analog Scale) was URLused, consisting of a 10 cm horizontal line, with one end representing “no pain” and the other end representing “worst possible pain.” Patients were asked to mark an “X” on the horizontal line to indicate their level of pain. The distance between the “no pain” end and the patient’s mark quantified the intensity of the pain they experienced.

For each of the following days, from Day 1 to Day 15, the questionnaire requires the patient to record the level of pain in the surgical area using the same scale. The questionnaire also includes a section for the patient’s identification code, consisting of a progressive number and the year of birth, in order to ensure anonymized data collection.

The questionnaire asked patients to report on the use of analgesics and the corresponding dosage immediately after surgery and on the first, second, third, fourth, fifth, sixth, seventh, and fifteenth days.

Each patient was recalled for a follow-up appointment one week after the procedure, during which the sutures were removed, and the surgical site was examined.

### 2.3. Statistical Analysis

Within each group, the patient was considered as the statistical unit. The data were analyzed using non-parametric tests due to the asymmetric distribution of some data sets (Shapiro–Wilk test: *p* < 0.05).

The power of this study was 80%. The alpha error was set at 0.05, and the Acceptable Difference % of the marginal of error was 15%. Calculations were performed using open source (URL accessed on 14 August 2024 https://www.benchmarksixsigma.com/calculators/sample-size-estimationproportion-data). The required number of implants was 27 for each group.

A Kruskal–Wallis test was used to assess the significance of differences in operative times between the various groups; both the absolute implant site preparation times and the relative times based on bone quality D1–D2 and D3–D4 were analyzed separately. Subsequently, a Dunn test was used for post hoc analyses. The Mann–Whitney test was used to assess the significance of differences in operative times related to bone quality D1–D2 and D3–D4 separately in the M group, in the D group, and in the P group. The Rcmdr software (version 2.9-2) was used for statistical analysis. A *p*-value less than 0.05 was used to reject a null hypothesis.

## 3. Results

In this study were enrolled 86 patients; 43 patients (30 women, 13 men; aged 45–70) received 84 implants placed with magnetodynamic surgery. The control group consisted of 43 patients (30 women, 13 men), each receiving at least 2 implants in contralateral sites, 1 prepared with the piezoelectric device and the other with a micromotor and dedicated drills, for a total of 150 implants (75 per technique). The patients who fell into the D1–D2 subgroup numbered 23 in each group. The patients who fell into the D3–D4 subgroup numbered 20 in each group.

### 3.1. Average Preparation Times

**Figure 1:**
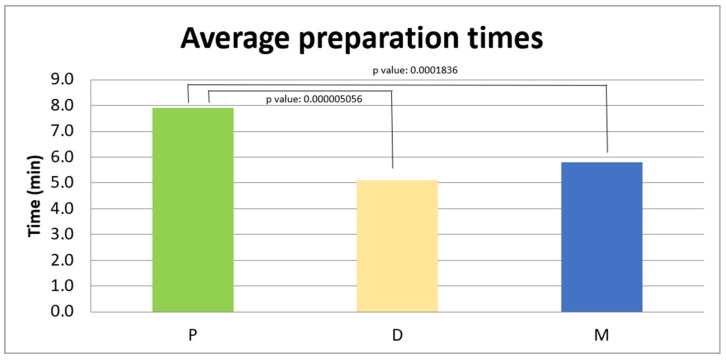
Average preparation times (in minutes) in the three groups: P 7.9 ± 2.1 min, D 6.0 ± 2.4 min, and M 6.4 ± 2.5 min.

The analyzed sample consisted of 43 patients for each group. The data are presented as the mean ± standard deviation. The average implant site preparation time in group P was 7.9 ± 2.1 min (95% CI: 7.21–8.50), in group D it was 6.0 ± 2.4 min (95% CI: 5.22–6.69), and in group M it was 6.4 ± 2.5 min (95% CI: 5.62–7.18), as shown in Figure 1.

A Kruskal–Wallis test was conducted to assess the presence of differences between the three groups regarding the average preparation times with the three methods.

The analysis showed a significant difference between the three groups (*p* < 0.01).

A post hoc analysis was conducted to identify specific differences between the M, D, and P groups.

In the comparison between the M and D groups, the *p*-value was 0.4111, higher than the significance threshold (*p* > 0.05), indicating that there is no significant difference between the two groups.

In the comparison between the M and P groups, the *p*-value was 0.0001836, lower than the set significance threshold, indicating that there is a statistically significant difference between the two groups.

In the comparison between the D and P groups, the *p*-value was 0.0000005056, much lower than the significance threshold, highlighting a statistically significant difference between the two groups.

#### 3.1.1. Average Preparation Times for D1–D2 Bone

**Figure 2:**
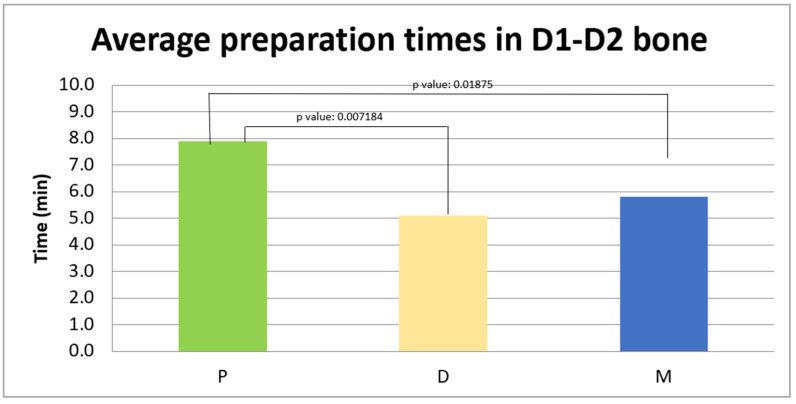
Average preparation times (in minutes) in D1–D2 bone in the three experimental groups: P 7.8 ± 1.7 min, D it 6.7 ± 2.5 min, and M 7.0 ± 3.1 min.

The analyzed sample consists of 23 patients for each group. The data are presented as the mean ± standard deviation: in group P, the average preparation time was 7.8 ± 1.7 min, in group D it was 6.7 ± 2.5 min, and in group M the average was 7.0 ± 3.1 min, as shown in Figure 2. A Kruskal–Wallis analysis was used to determine if there were statistically significant differences between the three groups in the average preparation times in the D3 density bone.

The analysis revealed a statistically significant difference between the three groups (*p* = 0.01352).

In the comparison between the M group and the D group, the *p*-value was 0.7582, indicating that there is no statistically significant difference between the data collected.

In the comparison between the P group and the D group, the *p*-value was 0.007184, indicating the existence of a statistically significant difference between the two groups.

In the comparison between the M group and the P group, the *p*-value was 0.01875, indicating the existence of a statistically significant difference between the two groups as the *p*-value was below the significance threshold.

#### 3.1.2. Average Preparation Times for D3–D4 Bone

**Figure 3:**
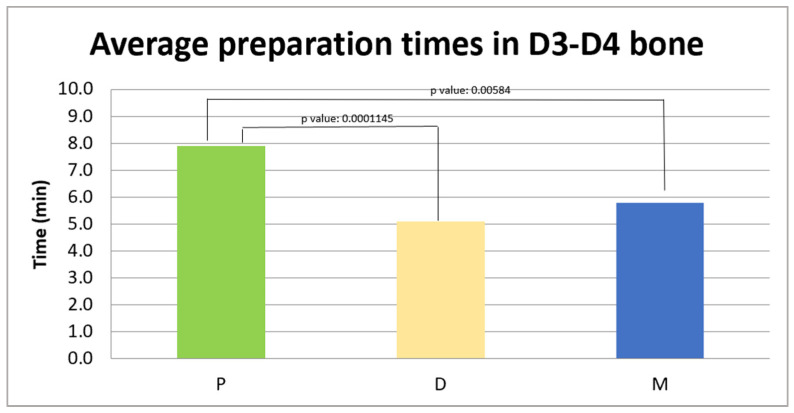
Average preparation times (in minutes) in D3–D4 bone in the three experimental groups: P 7.9 ± 2.5 min, D 5.1 ± 2.1 min, and M 5.8 ± 1.7 min.

The analyzed sample consists of 20 patients for each group. The data are presented as the mean ± standard deviation: in group P, the average preparation time was 7.9 ± 2.5 min, in group D it was 5.1 ± 2.1 min, and in group M it was 5.8 ± 1.7 min, as shown in Figure 3.

A Kruskal–Wallis analysis was conducted to determine if there were statistically significant differences between the three groups in the average preparation times in the D3–D4 density bone.

The analysis revealed a statistically significant difference between the three groups (*p* < 0.01).

In the comparison between the M and D groups, the *p*-value was 0.2511, higher than the significance threshold (*p* > 0.05), indicating that there is no significant difference between the two groups.

In the comparison between the M and P groups, the *p*-value was 0.00584, lower than the set significance threshold, indicating that there is a statistically significant difference between the two groups.

In the comparison between the D and P groups, the *p*-value was 0.0001145, lower than the significance threshold, highlighting a statistically significant difference between the two groups.

### 3.2. Average Preparation Times in the Magnetic Mallet Group for D1–D2 and D3–D4 Bone

**Figure 4:**
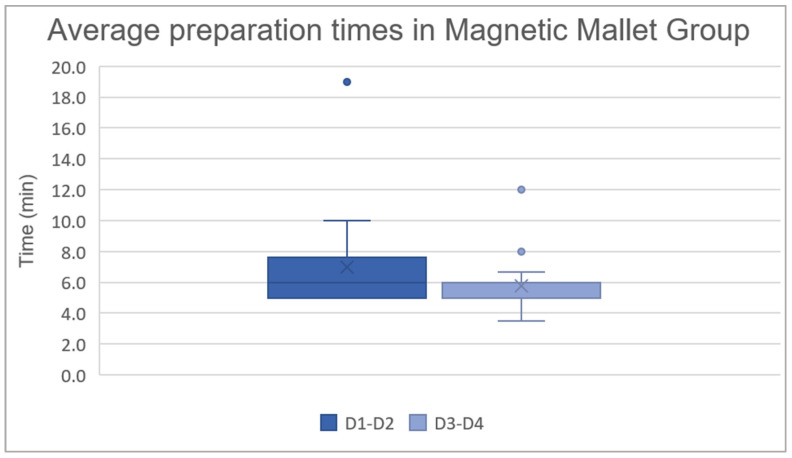
Average preparation times (in minutes) in the Magnetic Mallet group: D1–D2 7.0 ± 3.1 min and D3–D4 5.8 ± 1.7 min.

The analyzed sample consists of 23 patients in the D1–D2 group and 20 patients in the D3–D4 group. The data are presented as the mean ± standard deviation: in the D1–D2 group the average preparation time was 7.0 ± 3.1 min, and in the D3–D4 group it was 5.8 ± 1.7 min. A Mann–Whitney test was used to investigate whether there is a statistically significant difference between the preparation times of the implant site using the Magnetic Mallet in D1–D2 and D3–D4 bone. The analysis revealed a z-score of 2.0409 and a *p*-value of 0.04136, indicating a statistically significant difference between the two groups, as shown in the graph below, with a probability of less than 5% that this difference is due to chance (Figure 4).

### 3.3. Average Preparation Times in Drill Group for D1–D2 and D3–D4 Bone

The analyzed sample consists of 23 patients in the D1–D2 group and 20 patients in the D3–D4 group. The data are presented as the mean ± standard deviation: in the D1–D2 group the average preparation time was 6.7 ± 2.5 min, and in the D3–D4 group it was 5.1 ± 2.1 min.

**Figure 5:**
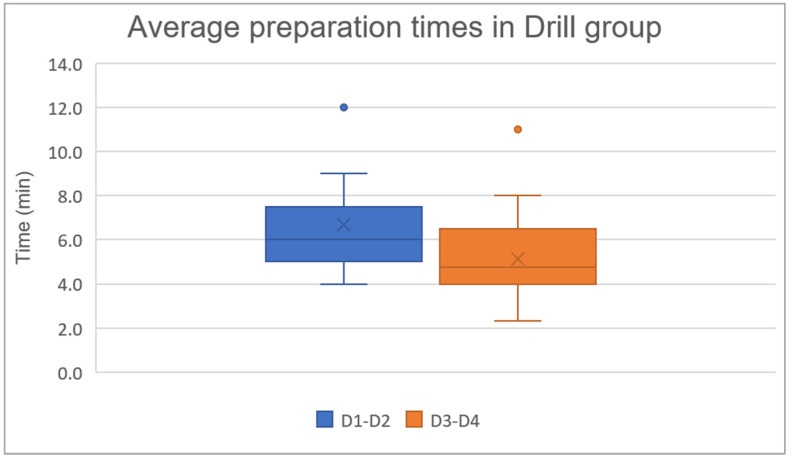
Average preparation times in drill group: D1–D2 6.7 ± 2.5 min and D3–D4 5.1 ± 2.1 min.

A Mann–Whitney test was used to investigate whether there is a statistically significant difference between the preparation times of the implant site using drills mounted on the micromotor in the D1–D2 and D3–D4 bone. The analysis revealed a *p*-value of 0.01778, indicating a statistically significant difference between the two groups (Figure 5).

### 3.4. Average Preparation Times in Piezodevice Group for D1–D2 and D3–D4 Bone

**Figure 6:**
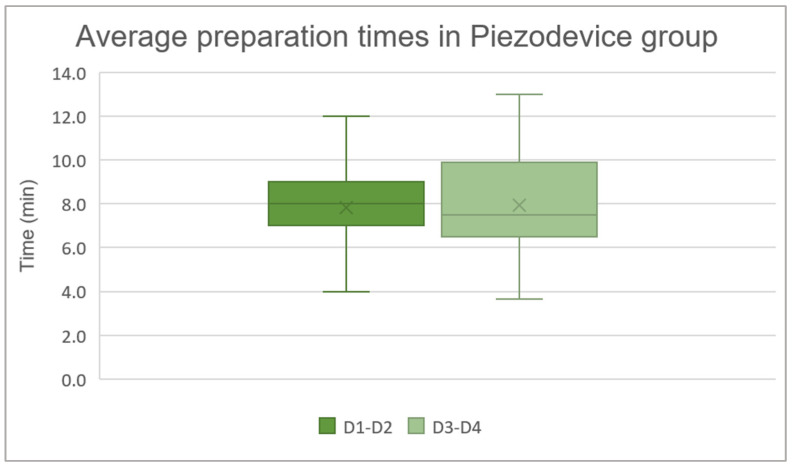
Average preparation times in Piezodevice group: D1–D2 7.8 ± 1.7 min and D3–D4 7.9 ± 2.5 min.

The analyzed sample consists of 23 patients in the D1–D2 group and 20 patients in the D3–D4 group. The data are presented as the mean ± standard deviation: in the D1–D2 group the average preparation time was 7.8 ± 1.7 min, and in the D3–D4 group it was 7.9 ± 2.5 min. A Mann–Whitney test was used to investigate whether there is a statistically significant difference between the preparation times of the implant site using the Piezodevice in the D1–D2 and D3–D4 bone. The analysis revealed a *p*-value of 0.9124, indicating that there is no statistically significant difference between the two groups (Figure 6).

Regarding the VAS reported by patients during the procedure, a graph was created based on the recorded data, showing the trend of VAS values over time for the three groups.

The VAS values showed a progressive decrease over time in all groups. At T0, mean VAS scores were 4.05 [95% CI: 3.58–4.52] for F, 1.28 [95% CI: 0.93–1.62] for M, and 2.51 [95% CI: 2.12–2.91] for P. At T1, values decreased to 3.58 [95% CI: 3.09–4.08] for F, 1.91 [95% CI: 1.46–2.35] for M, and 2.09 [95% CI: 1.61–2.57] for P. At T2, VAS values further declined, with F 2.72 [95% CI: 2.17–3.27], M 0.93 [95% CI: 0.59–1.27], and P 1.47 [95% CI: 1.05–1.88]. At T3, scores reached F 2.28 [95% CI: 1.72–2.84], M 0.14 [95% CI: 0.02–0.30], and P 1.02 [95% CI: 0.60–1.45]. A continued reduction was observed at T4 (F 1.67 [95% CI: 1.15–2.20], M 0.05 [95% CI: 0.05–0.14], P 0.70 [95% CI: 0.36–1.04]) and T5 (F 0.86 [95% CI: 0.45–1.27], M 0.26 [95% CI: 0.06–0.45], P 0.12 [95% CI: 0.00–0.24]).

**Figure 7:**
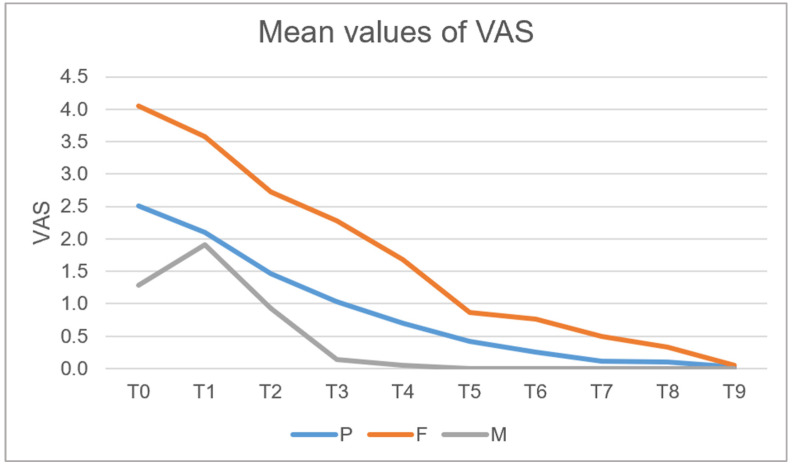
Mean values of VAS in the three examined groups.

At T6, VAS values were 0.42 [95% CI: 0.17–0.67] for F, 0.77 [95% CI: 0.41–1.13] for M, and 0.26 [95% CI: 0.06–0.45] for P. At T7, pain levels were minimal for all groups, with F 0.49 [95% CI: 0.17–0.81], M 0.12 [95% CI: 0.00–0.24], and P 0.09 [95% CI: 0.00–0.18]. At T8, values remained low (F 0.12 [95% CI: 0.00–0.24], M 0.33 [95% CI: 0.12–0.53], P 0.09 [95% CI: 0.00–0.18]). Finally, at T9, VAS scores were 0.02 [95% CI: 0.02–0.07] for all of three groups (Figure 7).

A preliminary qualitative analysis suggests the existence of a difference between the three surgical techniques examined.

**Figure 8:**
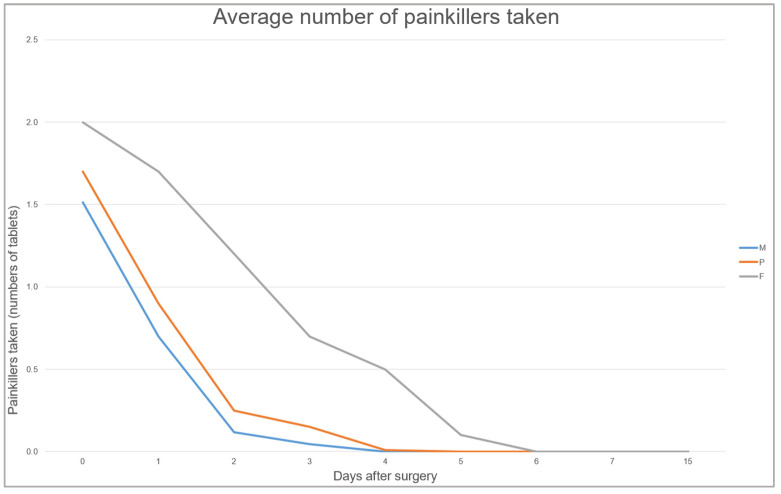
Average number of painkillers taken in the three experimental groups.

Regarding the average number of painkillers taken by patients during the procedure, a graph was created based on Excel data (Figure 8).

## 4. Discussion

Implant site preparation is a crucial phase for the long-term success of the implant fixture and is closely related to the primary stability that the implant will have [22,23]. Acting as conservatively and atraumatically as possible toward the surrounding bone tissue and soft tissues is currently one of the main objectives of implant surgery [1], in order to limit unwanted bone resorption and trauma. Preparation techniques can utilize different tools, each with a distinct mechanism of action that justifies their limitations and advantages. The results obtained in our experimental study highlighted statistically significant differences in the preparation of the implant site among the three examined preparation methods: the Magnetic Mallet, the Piezodevice, and the drills mounted on a slow-speed handpiece.

The statistical analyses showed that the average preparation times for the implant site with the Magnetic Mallet were intermediate compared to traditional surgery with drills and piezosurgery. The comparison between the Magnetic Mallet and the drills revealed that despite the drills operating with shorter average times, there is no statistically significant difference in preparation time, neither in absolute terms nor in relative terms based on the bone type, suggesting that both methods can be considered comparable in terms of speed of action. This finding cannot be compared with the existing literature due to the lack of studies on this subject, but similar findings have been observed in dental extraction procedures in the study conducted by Bennardo et al.: it was found that the Magnetic Mallet reduced the time needed for tooth extractions when compared to conventional instruments and piezosurgery [24].

From the perspective of optimizing not the surgical time but rather the number of surgical appointments, the use of instruments such as the Magnetic Mallet is of particular interest. For example, Crespi et al. (2021) demonstrated in their retrospective cone beam study that this device supports bone preservation and enables controlled bone augmentation [25].

Their paper suggests the application of magnetodynamic technology during split-crest procedures for horizontal ridge expansion. The authors also report that its use tends to facilitate immediate implant placements while ensuring long-term stability.

However, within the limits of the retrospective nature of this study, further studies are necessary to validate the findings of this study due to the limited number of studies available on this topic in the literature. Despite the similarity between the two groups in terms of preparation speed, the available literature has shown differences between the two methods regarding their effects on bone tissue [2,24,26].

Drills, although an established method for preparing the implant site, have some clinical disadvantages. The tissue subtraction caused by the use of rotating instruments can result in excessive bone removal [27], negatively impacting the precision of the implant site preparation and, consequently, the primary stability of the fixture [6]. Additionally, due to their high rotational speed, drills have been shown to increase the temperature at the surgical site [28], raising the risk of cellular necrosis and adversely affecting bone healing. As demonstrated by Raj et al. [5], the rotation speed of the drills, the pressure exerted on the handpiece by the operator, and the temperature of the irrigating solution are all critical variables to control during implant site preparation to prevent the temperature at the surgical site from exceeding the critical threshold of 47 °C for cellular necrosis; also the cortical thickness is a factor to consider for the increase in the temperature at the surgical site [29]. The Magnetic Mallet, on the other hand, compacts the bone rather than subtracting it, allowing for greater preservation of bone tissue and potentially improving the bone density and primary implant stability, in accordance with the study conducted by Antonelli et al. [30]. Additionally, both the mechanical trauma and the thermal trauma induced by the electromagnetic impulse on the bone tissue are significantly lower compared to the trauma caused by rotating instruments, considering that the contact between the tool and the tissue lasts only microseconds [12]. In the histological study by Schierano et al. [2], it was highlighted that the deposition of new bone tissue by osteoblasts is stimulated at the site treated with the Magnetic Mallet, and the inflammation at the surgical site is reduced.

The comparison between Magnetic Mallet and Piezodevice showed a statistically significant difference in both the average execution times for both the D2 and D3 bone. Piezosurgery, while recognized and used as a precise and safe technique [15], requires longer operative times compared to both rotating instruments and the Magnetic Mallet. The literature supports the use of ultrasonic instruments in situations where the preservation of vital structures is of primary importance as it operates at specific frequencies (24–36 kHz), allowing for selective cutting [8,18]. Bone surgery with piezoelectric instruments accelerates healing processes at the surgical site [31]; minimizes the necrosis of the bone tissue while supporting bone regeneration; enables a less traumatic [32], precise, and controlled surgery with micrometric cuts; and ensures better cleaning and visibility of the surgical site thanks to the cavitation effect [7]. However, the increase in surgical times [27,33] represents a limitation for the use of this instrument, and from this perspective, the Magnetic Mallet may prove to be an efficient alternative to the Piezodevice in cases where reduced operative time is required without compromising the quality of the procedure.

In this study, the bone quality significantly influenced the operative times in the group treated with the Magnetic Mallet and in the group treated with the traditional technique, with a statistically significant difference between the D1–D2 and D3–D4 groups. D2 bone, according to Misch’s classification [21], is more resistant to the penetration of instruments because it is rich in cortical bone and has dense medullary tissue, which may explain why a longer action time was required compared to the D3 bone, which has a higher medullary component. The electromagnetic impulse of the Magnetic Mallet has been shown to compact bone laterally in sites with poor bone quality [34], potentially providing a clinical advantage in improving implant stability in situations where the bone quality is not adequate to ensure proper primary stability [35]. However, further in vivo studies are needed to explore this advantage. The instrument transfers a high-intensity controlled force to a well-localized area of the bone tissue, limiting the risk of force propagation throughout the maxillofacial complex and reducing the risk of unwanted fractures and complications [36]. However, the action of the magnetodynamic instrument on dense bone may not represent an advantage or an alternative to traditional surgery, as it has been shown to potentially lead to unwanted cortical fractures [13].

From a practical standpoint, the experience of the operators involved in this study, together with the feedback obtained during the initial use of the technologies, highlighted several noteworthy aspects regarding the devices and their respective learning curves. Historically, the most widely adopted system has been the drill-based approach. Drills are generally considered highly ergonomic instruments for clinicians with a reasonable level of implantology training. Depending on the manufacturer, contemporary drill systems typically provide a straightforward sequence of burs in ascending diameters, thereby simplifying the osteotomy preparation.

Nevertheless, inexperienced operators may still encounter critical challenges when first approaching an implant site. For example, it is relatively easy to deviate from the intended drilling axis, overprepare the osteotomy, or cause the overheating of the bone. Compared with drills, piezoelectric and magnetodynamic devices appear to reduce these risks. However, these newer instruments present other practical limitations. In private practice, they require additional equipment beyond the implant motor or insertion unit. Moreover, even with experience, the operating time associated with piezoelectric devices tends to remain longer, which may be problematic in patients for whom the procedural speed is critical.

The Magnetic Mallet, on the other hand, is associated with a markedly longer learning curve, and its ease of use is strongly influenced by factors such as anatomical site accessibility and the patient’s mouth opening capacity.

Another aspect evaluated in our study concerned intraoperative and postoperative pain, measured using the Visual Analog Scale (VAS). In all the examined groups, a progressive decrease in perceived pain was observed in the postoperative days; however, the group treated with drills reported higher pain levels both intraoperatively and in the first few postoperative days, suggesting that drills may cause greater surgical trauma compared to the other two methods analyzed. This finding is consistent with the existing literature that correlates higher postoperative pain with the use of drills, not only due to the mechanical trauma induced on both soft and hard tissues [6] but also because of the characteristic of the Magnetic Mallet and piezosurgery.

The Magnetic Mallet demonstrated lower VAS values compared to the other groups, with pain completely alleviated by T4 (on the third postoperative day), suggesting not only greater postoperative comfort but also a reduced inflammatory response at the surgical site, as also highlighted by the literature [2]. Furthermore, patients treated with the Magnetic Mallet reported a lower average consumption of analgesic medications, indicating a more favorable postoperative course.

In addition to experiencing discomfort, many patients have difficulty tolerating prolonged operative times. This issue is particularly relevant in an increasingly aging population, as maintaining mouth opening and remaining in the same position for extended periods can be physically demanding. Moreover, clinicians themselves may experience reduced concentration during lengthy procedures or when required to alternate between different instruments.

## 5. Conclusions

To fully understand all the advantages of the Magnetic Mallet in the implant field compared to other surgical methods, further studies are required, both in vivo and in vitro, due to the current lack of studies in the literature.

Within the limitations of our study, based on the findings, the Magnetic Mallet offers a valuable balance, providing a comparable operational speed to traditional drills while concurrently yielding lower patient-reported pain scores (VAS). Conversely, while the Piezodevice also reduced pain compared to traditional drills, its significantly longer operative time may limit its efficiency.

Given the lack of studies in the literature comparing these three methods in the implant field, the future perspective of this study is to expand the cohort of enrolled patients and to continue data collection in order to improve the reliability of this study.

## Figures and Tables

**Table 1:** Exclusion criteria.

Category	Exclusion Criteria
Systemic Health	General medical contraindications to surgery or implant placement.
	Severe coagulation or immune system disorders (including compromised/weakened immune system).
	Uncontrolled diabetes.
	History of chemotherapy (within the past year) or radiotherapy (head and neck area).
	Current or previous treatment with intravenous amino bisphosphonates.
Local Conditions/Surgical Needs	Surgical sites presenting acute infection or purulent discharge.
	Cases requiring maxillary sinus regenerative surgery during implant fixture placement.
Patient Habits/Compliance	Patients with psychiatric disorders.
	Poor oral hygiene or parafunctional habits (e.g., severe bruxism).
	Patients with a history of drug or alcohol abuse.

## Data Availability

The original contributions presented in this study are included in the article. Further inquiries can be directed to the corresponding author.
